# Improving long-term kidney allograft survival by rethinking HLA compatibility: from molecular matching to non-HLA genes

**DOI:** 10.3389/fgene.2024.1442018

**Published:** 2024-10-02

**Authors:** Aprajita Mattoo, Ian S. Jaffe, Brendan Keating, Robert A. Montgomery, Massimo Mangiola

**Affiliations:** NYU Langone Transplant Institute, New York University Langone Health, New York, NY, United States

**Keywords:** eplet, PIRCHE-II, HLAMatchmaker, eplet registry, non-classical HLA, non-HLA genes, kidney transplant, kidney paired exchange

## Abstract

Optimizing immunologic compatibility in organ transplantation extends beyond the conventional approach of Human Leukocyte Antigen (HLA) antigen matching, which exhibits significant limitations. A broader comprehension of the roles of classical and non-classical HLA genes in transplantation is imperative for enhancing long-term graft survival. High-resolution molecular HLA genotyping, despite its inherent challenges, has emerged as the cornerstone for precise patient-donor compatibility assessment. Leveraging understanding of eplet biology and indirect immune activation, eplet mismatch calculators and the PIRCHE-II algorithm surpass traditional methods in predicting allograft rejection. Understanding minor histocompatibility antigens may also present an opportunity to personalize the compatibility process. While the application of molecular matching in deceased donor organ allocation presents multiple technical, logistical, and conceptual barriers, rendering it premature for mainstream use, several other areas of donor-recipient matching and post-transplant management are ready to incorporate molecular matching. Provision of molecular mismatch scores to physicians during potential organ offer evaluations could potentially amplify long-term outcomes. The implementation of molecular matching in living organ donation and kidney paired exchange programs is similarly viable. This article will explore the current understanding of immunologic matching in transplantation and the potential applications of epitope and non-epitope molecular biology and genetics in clinical transplantation.

## 1 Introduction

Historically, immunologic compatibility between transplant candidates and their solid organ donors has been determined by the level of antigen match at human leukocyte antigen (HLA) -A, -B and -DR. Despite significant technological improvements in the detection of HLA antibodies and HLA genotyping, organ compatibility is still determined by HLA antigen match at these loci.

According to the Organ Procurement and Transplantation Network (OPTN) there are a total of 103,829 patients awaiting transplantation in the United States as of May 2024, 86% of which are waiting for a kidney (Data OPTN, 2024). However, new waitlist registrations exceed transplants, and many more patients are never considered for transplant due to the shortage of organs available (Matas et al., 2023). Additionally, according to the OPTN annual data, between 2007 and 2019 the rate of re-transplantation of adult kidney candidates has been averaging around 14.3%. This is largely because, despite the tremendous progress made with improving short-term kidney graft survival (Sellarés et al., 2012), the median kidney allograft survival in the Unites States is still 11.2 years (Wu et al., 2017).

Therefore, between the influx of new transplant candidates, the rate of re-transplantation, and the disproportion between organ availability and transplant candidates, the waiting list is only expected to grow longer each year. Much effort has been focused on increasing the number of organs available for transplant through extended criteria, marginal, Hepatitis C positive, and other organs (Cecka, 2010). However, demand-focused interventions have been uncommon. While the overall burden of kidney disease cannot be tackled by the transplant community alone, we do have considerable ability to influence the sizeable need for kidney re-transplants. To do so, we must answer a fundamental question: how can long-term graft survival be significantly improved? The answer seems to be gravitating around optimizing the level of immunologic compatibility between recipient and donor.

It is important to understand that immunologic compatibility is not just a question of whether a recipient is compatible with a donor, but rather a question of “how much”. Conventional HLA antigen matching assumed that compatibility is strictly dependent on the number of shared HLA antigens. However, immunologic compatibility is a very dynamic process that involves much more than simple antigen match at HLA-A, -B and–DR.

When considering improving recipient/donor immunologic compatibility, one must consider two important aspects: improving the matching strategy for classical HLA genes and determining the immunogenic role of non-HLA genes. In this review, we will summarize the most recent scientific advancements in understanding the role of HLA and non-HLA genes in transplantation and outline potential paths forward for advancements that are ready to enter the clinic.

## 2 The limitations of conventional HLA antigen matching and the need for a new matching strategy

Immunologic compatibility in organ transplantation is primarily dependent on the degree of HLA match between donor and recipient transplant pairs. Studies have consistently demonstrated that the greater the degree of Class I and Class II HLA antigen mismatch, the greater the likelihood of worse allograft survival in both living donor and deceased donor kidney transplantation. This relationship is attributed to HLA antigen mismatches associated with the development of anti-HLA donor-specific antibodies (DSA) in recipients (Krummey et al., 2022). The presence of *de novo* DSA (dnDSA) leads to an increased risk for acute and chronic antibody mediated rejection (AMR)—the principal cause of long-term allograft failure in kidney transplantation (Sellarés et al., 2012). The critical importance of HLA antigen matching has led to the worldwide utilization of deceased donor organ allocation systems that prioritize donor-recipient transplants pairs by the fewest possible HLA antigen mismatches at HLA-DR (and HLA-A and -B with prioritization for 0-ABDR mismatches) (Wu et al., 2017). Many deceased donor organ allocation systems also apply the calculated panel reactive antibodies percentage (% cPRA) in their algorithms. The % cPRA represents the percentage of the donor pool against whom the recipient has unacceptable antibodies and is based on the frequencies of HLA antigens in the donor population (Cecka, 2010).

Over the past 50 years, HLA typing has evolved from serology-based methods to high-resolution molecular genotyping assays that have now identified over 20,000 different HLA alleles (Krummey et al., 2022). Accurate identification of the HLA antigens expressed by a donor is essential to correctly identify a recipient’s preformed DSAs and optimize donor-recipient compatibility. Compatibility optimization minimizes a recipient’s exposure to immunogenic HLA antigens, thus decreasing the risk of dnDSA formation and AMR, and promotes long term graft survival. Moreover, precise identification of donor’s HLA antigens and their recipient’s preformed DSA allows for strategies and therapies to be employed pre- and post-transplant that may reduce the risk of rejection when crossing the HLA barrier is unavoidable (Noble et al., 2023).

The introduction of the molecular HLA genotype requirement in UNOS and the expansion to HLA-C, -DQA1, -DQB1, and -DPB1 greatly reduced the number of unexpected positive crossmatches and improved allocation. However, the introduction of molecular HLA genotyping did not come without challenges. Before the introduction of molecular genotyping, the UNOS matching algorithm was challenged by lack of specificity and the difficulty with resolving the serological splits of certain HLA antigens (i.e., separate antigens with the capability of having different antibodies directed against them that were originally believed to be a single common antigen, such as A23 and A24 being splits of A9). After the introduction of molecular HLA genotyping, the challenge was to convert molecular typing into serological equivalency (the backbone of the UNOS algorithm). Eventually, to facilitate the generation of serological equivalents, a molecular-to-serology conversion script was developed (Kaur et al., 2018). From this script, equivalency tables were generated, and these are used by UNOS to determine recipient/donor equivalency and for equivalency with unacceptable antigens (Table 1).

**TABLE 1:** Sample UNOS serological equivalency table.

HLA allele	Serological equivalent	Recipient unacceptable antigen	Donor equivalent unacceptable antigen(s)
B*14:01	B64	B64	B64, B*14:01
B*15:01	B62	B62	B62, B*15:01, B*15:04, B*15:06, B*15:07, B*15:20. B*15:27
B*40:02	B61	B61	B61, B*40:02, B*40:03, B*40:04, B*40:06
DRB1*03:02	DR18	DR18	DR18, DRB1*03:02, DRB1*03:03
DQB1*03:01	DQ7	DQ7	DQ7, DQB1*03:01, DQB1*03:19

**FIGURE 1:**
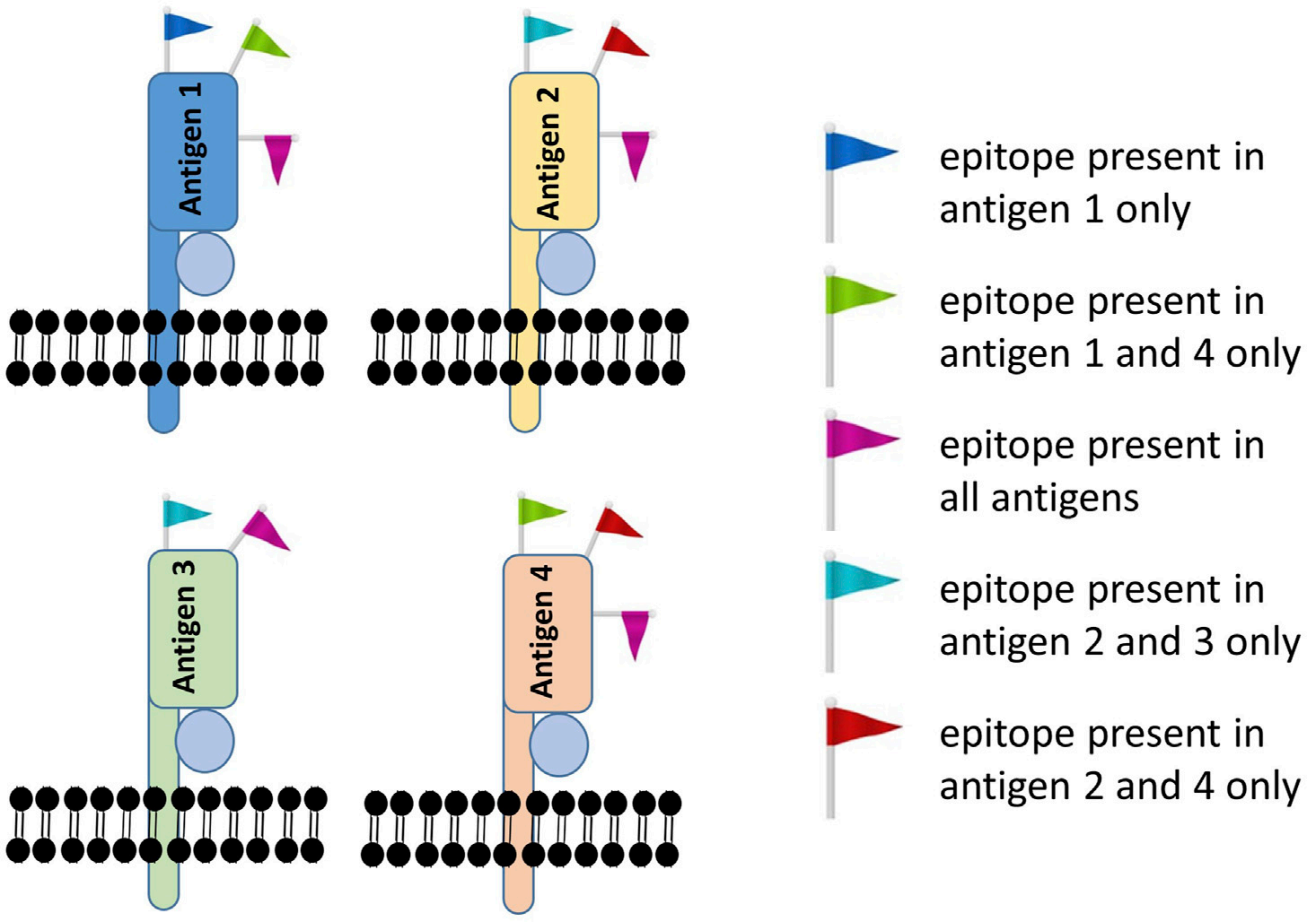
Private versus public epitope motifs. Each HLA antigen (represented by example antigens 1–4) possesses multiple epitope motifs that represent anti-HLA antibody binding sites. Epitopes can be private (such as the blue epitope, present on antigen 1 only) or public (such as the pink epitope, present on all antigens).

Since then, molecular genotyping has evolved from low-resolution, to high-resolution and all the way to genome-wide genotyping (Picascia et al., 2016). Low-resolution (2-digit) HLA genotyping (aka antigen-level resolution) is a fast and reliable genotyping method but has its limitations. Typically, it describes a group of HLA alleles having a high degree of homology in their amino-acid sequence. Within the same antigenic group, HLA alleles are molecularly similar (high degree of homology) but differ at one or more amino-acid and are not necessarily the same in terms of serological motif. Anti-HLA antibodies do not bind the whole HLA antigen, but to specific patches of amino acids known as HLA epitopes. HLA epitopes describe serological motifs that are shared by few or many HLA antigens. Some epitopes are specific to one or a few HLA antigens (private epitope motifs), while others are shared by many HLA antigens (public epitope motifs) (Figure 1).

Based on the technology in use, low-resolution genotyping of donors runs the risk of excluding an entire broad HLA antigen group due to the presence, in the recipient serum, of anti-HLA antibodies that may not necessarily react with all the different alleles within the same HLA group. Table 2 is an example of HLA motifs expressed by some HLA-A2 and HLA-DR4 alleles present in the Luminex^®^ HLA antibody testing panel. As shown in Table 2, the 149TAH, 145HT, 151AHE and 152E motifs are uniquely expressed by the HLA-A*02:03 allele, whereas all the other HLA-A2-motifs are expressed by different combinations of HLA-A2 antigens. Similarly, the Class II HLA-DR4 motifs are expressed by a combination of different alleles, with exclusion of 57S (expressed only by DRB1*04:05), 70D, 70DA, and 71E (expressed only by DRB1*04:02), and 74E (expressed only by DRB1*04:03). These examples clearly demonstrate how the identification of the exact HLA allele can be instrumental when determining recipient/donor compatibility. In fact, an antibody targeting the 149TAH motif would only rule-out donors expressing the A*02:03, but not donors expressing the other common A2 antigens. Similarly, an anti-57S antibody would only rule-out donors expressing the DRB1*04:05 alleles and not exclude donor with other DRB1*04 alleles.

**TABLE 2:** HLA motifs expressed by alleles of the A2 and DR4 antigens present in the Luminex^®^ HLA antibody testing panel.

Antigen	Allele	Antibody-verified motifs			Other (provisional) motifs (R)										
A2	A*02:01	145KHA		150AAH			95V		149AH				151AHV				152V		156L	
	A*02:02	145KHA		150AAH	43	95L			149AH				151AHV				152V			156WA
	A*02:03		149TAH				95V	145HT			151AHE				152E					156WA
	A*02:05	145KHA		150AAH	43	95L			149AH				151AHV				152V			156WA
	A*02:06	145KHA		150AAH			95V		149AH				151AHV				152V		156L	

Antigen	Allele	Antibody-verified motifs			Other (Provisional) motifs															
DR4	DRB1*04:01		67LQ			70QT	57D	57DA	70Q	70QA	70QK		71K			74A			86G	
	DRB1*04:02			70D	70DA		57D	57DA						71E		74A		85VV		86V
	DRB1*04:03		67LQ			70QT	57D	57DA	70Q	70QA		70QRA			71R		74E	85VV		86V
	DRB1*04:04		67LQ			70QT	57D	57DA	70Q	70QA		70QRA			71R	74A		85VV		86V
	DRB1*04:05	57S	67LQ			70QT			70Q	70QA		70QRA			71R	74A			86G	

On the other hand, high-resolution (4-digit) HLA genotyping describes individual HLA alleles, down to a single amino-acid polymorphism (SAP) in their protein sequence (Cereb et al., 2015). Although this methodology greatly improves compatibility assessment, it presents its own set of challenges, as thousands of distinct HLA alleles can be identified. In fact, allele-level donor-recipient HLA matching can result in an overall increase in the level of mismatch between donor-recipient pairs. However, the SAPs between two alleles are not always serologically relevant, either because the polymorphism is not exposed in the molecular surface or because the recipient’s antibodies do not target that polymorphic position (Kosmoliaptsis et al., 2014). However, it is worth mentioning that unexposed amino acids can still meaningfully affect the electrostatic conformation of the antigen and thus antibody binding. Moreover, as more HLA alleles are discovered, the challenge of mapping the serological motifs expressed in these new antigens and the equivalency to previously discovered alleles still remains. HLA specialists and geneticists frequently update the molecular and serological tables used by UNOS and several free-to-use online tools such as Allele Frequency Net (www.allelefrequencies.net/) are available to guide users through common and uncommon HLA alleles.

**FIGURE 2:**
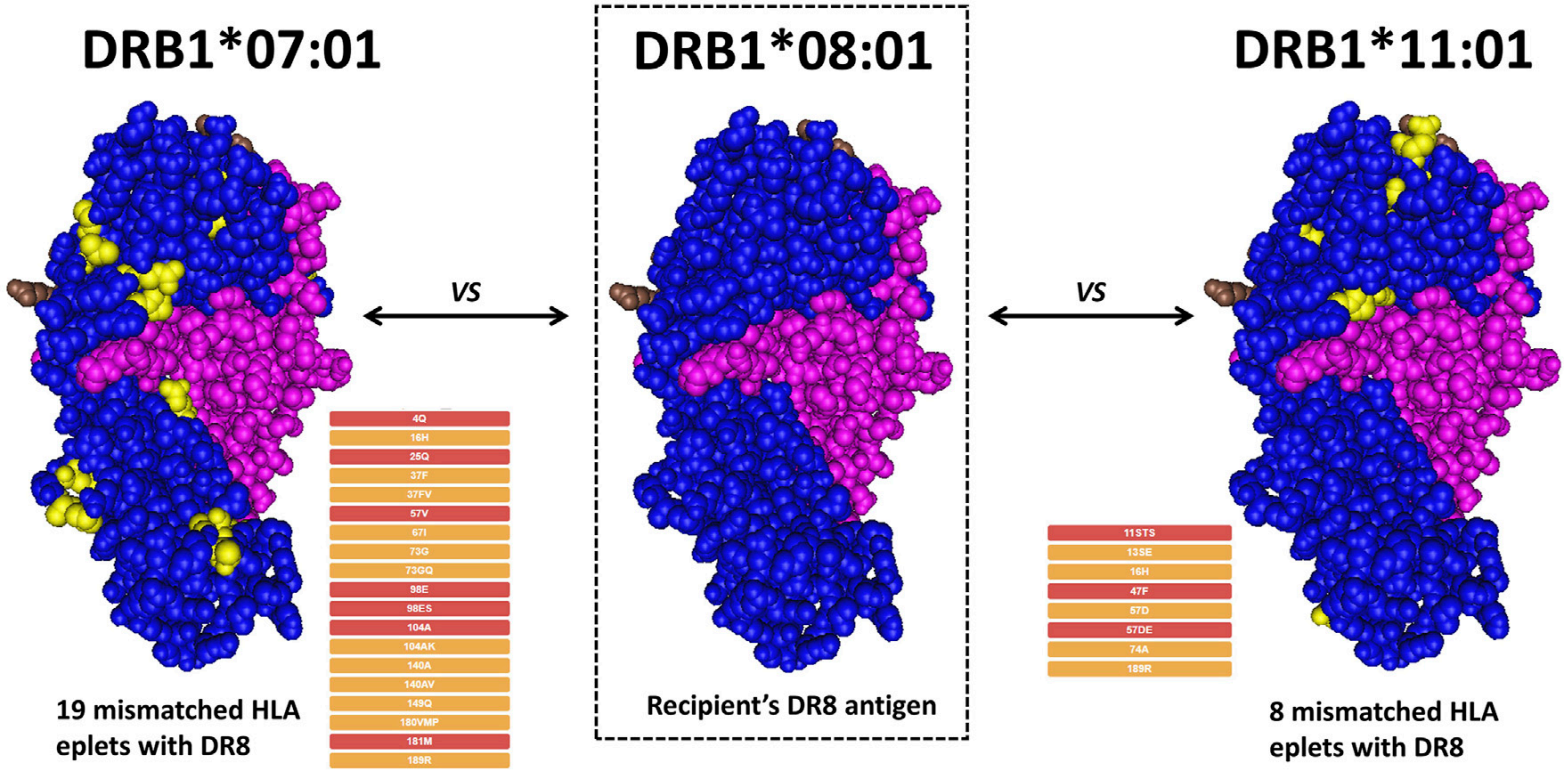
Example of eplet mismatch counting between different DRB1 alleles. A hypothetical recipient expressing DRB1*08:01 is compared with two potential donors. Under the traditional antigen match score, both donors would be considered to have the same degree of match. However, a donor with DRB1*01:01 would represent a 19-eplet mismatch, while a donor with DRB1*11:01 would represent an 8-eplet mismatch, corresponding to a much lower immunogenic risk.

Despite all the challenges that molecular HLA genotyping may pose as compared to conventional HLA antigen matching, it has become very evident that precise patient and donor compatibility assessment requires a molecular approach. Regardless of the genotyping method and the level of resolution, comparing patient and donor HLA antigens does not accurately describe the immunological differences of their HLA antigens. It is important to recognize that the numerical HLA nomenclature does not describe molecular “proximity” and does not always correlate with the serological motifs. For instance, HLA-A*01:01 is numerically closer to HLA-A*02:01 than HLA-A*80:01, but the eplet (motif) mismatch is 92.7% higher between A1-A2 (30 mismatched eplets) than between A1-A80 (11 mismatched eplets). Two potential donors with the same amount of antigen mismatch can easily be significantly different by molecular match. As shown in Figure 2, if the DRB1*08:01 antigen of a putative recipient is compared to the DRB*07:01 or the DRB1*11:01 of two putative donors, it is easy to demonstrate that the molecular match is higher between DR8 and DR7 (19 eplet mismatches in 1 single molecule) as compared to DR8 and DR11 (8 eplet mismatches in 1 single molecule).

Moreover, anti-HLA antibodies target serological motifs that can be present in hundreds of HLA antigens and yet be missing in one or few allelic variations of the same antigen group. On the other end, no matter the level of molecular resolution, comparing HLA alleles does not address the fact that certain molecular mismatches could be more immunogenic than others. The deleterious effect of pre-formed and *de novo* Class II DSA has been known for years (Wu et al., 2013; Caillard et al., 2017; Gniewkiewicz et al., 2023). We know now that the amount of molecular HLA mismatches at HLA-DR and-DQ is a strong risk predictor for the development of dnDSAs and allograft rejection (Wiebe et al., 2013). Interestingly, the same deleterious effect and the same risk prediction does not necessarily hold for pre-formed or *de novo* Class I DSAs (Hirai et al., 2014; Senev et al., 2020). In turn, this seems to indicate that not all molecular mismatches are created equal and that some mismatches are more dangerous than others.

Despite all the challenges that molecular HLA genotyping may pose, it is a fact that HLA compatibility is accurately established only when recipient and donor are compared based on the molecular differences in their HLA antigens. By comparing graft survival in the past four decades, advancements in immunosuppression regimens have significantly improved short-term graft survival. However, the same improvements are not translating into greater organ longevity and conventional HLA antigen matching has not impacted long-term graft survival. In turn, graft loss and patient reentry into the waiting list not only poses a new immunological challenge to find another compatible donor (especially when dnDSA have been developed) but also continues to weigh on the disparity between patient awaiting organs and available organs. Improving our matching strategies can improve long-term survival, but also be beneficial to highly sensitized kidney patients. Given the degree of pre-formed HLA antibodies, candidates with a cPRA >90% are proportionally unlikely to find an HLA compatible donor in either the deceased or the living donor organ pools. It is estimated that a 90% cPRA patient has a likelihood of finding a compatible donor that is 1/10th that of an unsensitized patient. The likelihood decreases to 1/10,000th for a patient with a 99.99% cPRA. Therefore, patients who are very highly sensitized are significantly disadvantaged and wait significantly longer before receiving an organ offer, even with increased priority under the new Kidney Allocation System (Schinstock et al., 2019). For highly sensitized patients, accurate identification of the molecular antibody motifs and determination of molecularly acceptable mismatches HLA allele may increase the donor pool and improve the probability of transplant. In 1989, the Eurotransplant system launched the “Acceptable Mismatch” program with the aim of providing a transplant avenue for highly sensitized patients (Heidt et al., 2019). This program identifies acceptable mismatched HLA alleles and is entirely based on molecular recognition of HLA antibody motifs (Heidt et al., 2021). By 2020, this initiative resulted in the successful transplant of 1790 highly sensitized patients (Heidt et al., 2021). Therefore, a new approach to the current paradigm of HLA matching is much needed. Recent advancement in molecular genotyping and the introduction of molecular HLA compatibility is the foundation for a new matching strategy.

## 3 The introduction of molecular HLA matching

**FIGURE 3:**
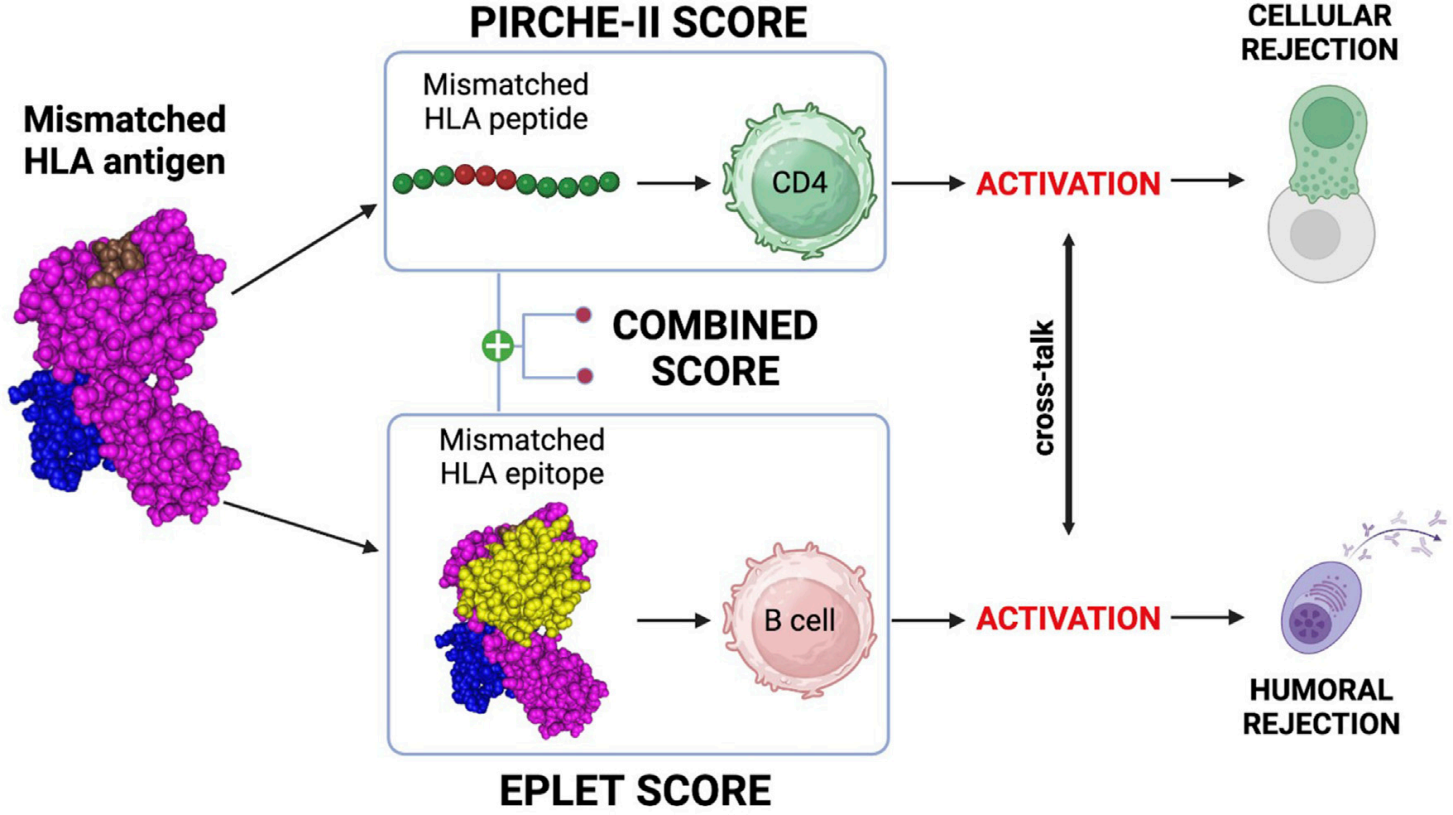
Molecular mismatch, T/B cells and molecular mismatch calculation. mismatched HLA antigen provides immunogenic peptides to CD4 T cells and epitopes to B cells. Activation of these cells and crosstalk can lead to generation of a primary, donor-specific alloimmune response. PIRCHE-II can be used to estimate the risk for CD4 activation and the eplet mismatch load can be used to estimate the risk of B cell activation. Both are powerful biomarkers for rejection and the combined T/B cell score can help to correctly identify the rejection risk. Created with BioRender.com.

More than antigen or allele HLA mismatch, the degree of molecular HLA match correlates with decreased allograft rejection (Kim et al., 2023). Donor-derived, mismatched HLA antigens provide immunogenic HLA peptides to CD4 T cells and immunogenic HLA epitopes to B cells (Figure 3).

Primary alloimmune responses to donor-derived mismatched T cell peptides and B cell epitopes are responsible for both cellular and humoral rejection (Senev et al., 2022; Meneghini et al., 2020). Mismatched HLA peptides are recognized by T cells by direct, semi-direct, and indirect pathways (Siu et al., 2018). The mode, duration and intensity of the allorecognition influences our ability to mediate early and late allograft rejection and will influence long-term graft outcomes (Siu et al., 2018). As shown in Figure 3, indirect CD4 T cell allorecognition and activation of the cellular response can be predicted using the Predicted Indirectly ReCognizable HLA Epitopes (PIRCHE-II) algorithm (Geneugelijk and Spierings, 2020). The PIRCHE-II score is a biomarker of indirect CD4 T cell activation, which is crucial for the development of both cellular and humoral responses (Bernard et al., 2005). B cells can recognize mismatched HLA epitopes through their B cell receptor (BCR). This recognition constitutes the first of two signals essential for maturation into plasma cells and production of epitope-specific antibodies (Ellison et al., 2023). The second signal is provided by cognate interaction with follicular helper T cells (TFH) activated by the same antigen (Ellison et al., 2023). This T/B cell cooperation mechanism is called Linked Recognition (Santos et al., 2024) and is an essential step of B cell immunity in solid organ transplantation (Ellison et al., 2023). As shown in Figure 3, the eplet mismatch load can be calculated with the Eplet Registry Calculator (https://www.epregistry.com.br/calculator/index.html) and represents a powerful biomarker of allograft rejection (Jackson and Pinelli, 2021; Lim et al., 2018). In virtually every organ, as we will explore, PIRCHE-II and eplet mismatch are individually powerful predictive biomarkers for the development of HLA antibodies and allograft rejection. Moreover, combining both T and B cell risk algorithms (Figure 3) has been shown to provide an even more accurate measurement of the risk for allograft rejection (Mangiola et al., 2022; Ellison et al., 2023; Santos et al., 2024). Although specific risk cut-offs for PIRCHE-II and eplet mismatch have yet to be clearly defined, the relevance of molecular compatibility in transplant immunology is not in question.

### 3.1 PIRCHE-II score

**FIGURE 4:**
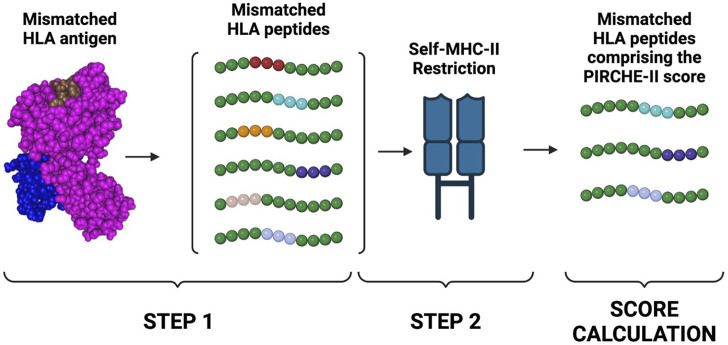
PIRCHE-II score calculation. The PIRCHE-II algorithm estimates the probability of indirect T cell activation. The algorithm will first determine which non-self peptides can be derived from a mismatched HLA antigen. PIRCHE-II will then determine which of the mismatched peptides is predicted to be bound by self-MHC II molecules (MHC-restriction). The score is then calculated by summing peptides derived from the mismatched HLA donor’s antigens that can be restricted by the Class II HLA-DR recipient’s antigens. Created with BioRender.com.

Several studies have established the importance of T cell recognition of non-self HLA epitopes in stimulating B cells and the generation of highly specific HLA antibodies (Sauvé et al., 2004; Lovegrove et al., 2001; Conlon et al., 2012). This can occur by a direct pathway, which involves CD4 T cells recognizing intact HLA alloantigen on the surface of donor antigen-presenting cells (APCs), or by an indirect pathway, which involves the recognition of processed peptides restricted by self-MHC Class II expressed on the surface of recipient APCs (Conlon et al., 2012). Post-transplant, indirect allo-presentation is the pathway with the longest and strongest duration (Siu et al., 2018). To estimate the risk of indirect allo-presentation, the Predicted Indirectly ReCognizable HLA Epitopes (PIRCHE-II) algorithm was developed by Geneugelijk and Spierings (2020). Based on predicted binding affinity of differing-from-self, donor-derived HLA peptides to recipient HLA Class II molecules, this algorithm can estimate the number of mismatched HLA peptides that can potentially activate CD4 T cells through the indirect pathway (Figure 4) (Geneugelijk et al., 2015).

Although there are numerous technical challenges undergoing refinement with iterative improvements to the PIRCHE-II algorithm (Geneugelijk and Spierings, 2020), PIRCHE-II has demonstrated correlation with graft rejection and failure across several organ and donor types (Mangiola et al., 2022; Lachmann et al., 2017; Geneugelijk et al., 2018; Meszaros et al., 2020). PIRCHE-II scores for HLA Class II antigens have also been associated with increased T-cell mediated rejection (TCMR) risk (Senev et al., 2022; Betjes et al., 2022).

### 3.2 Eplet mismatch load

**FIGURE 5:**
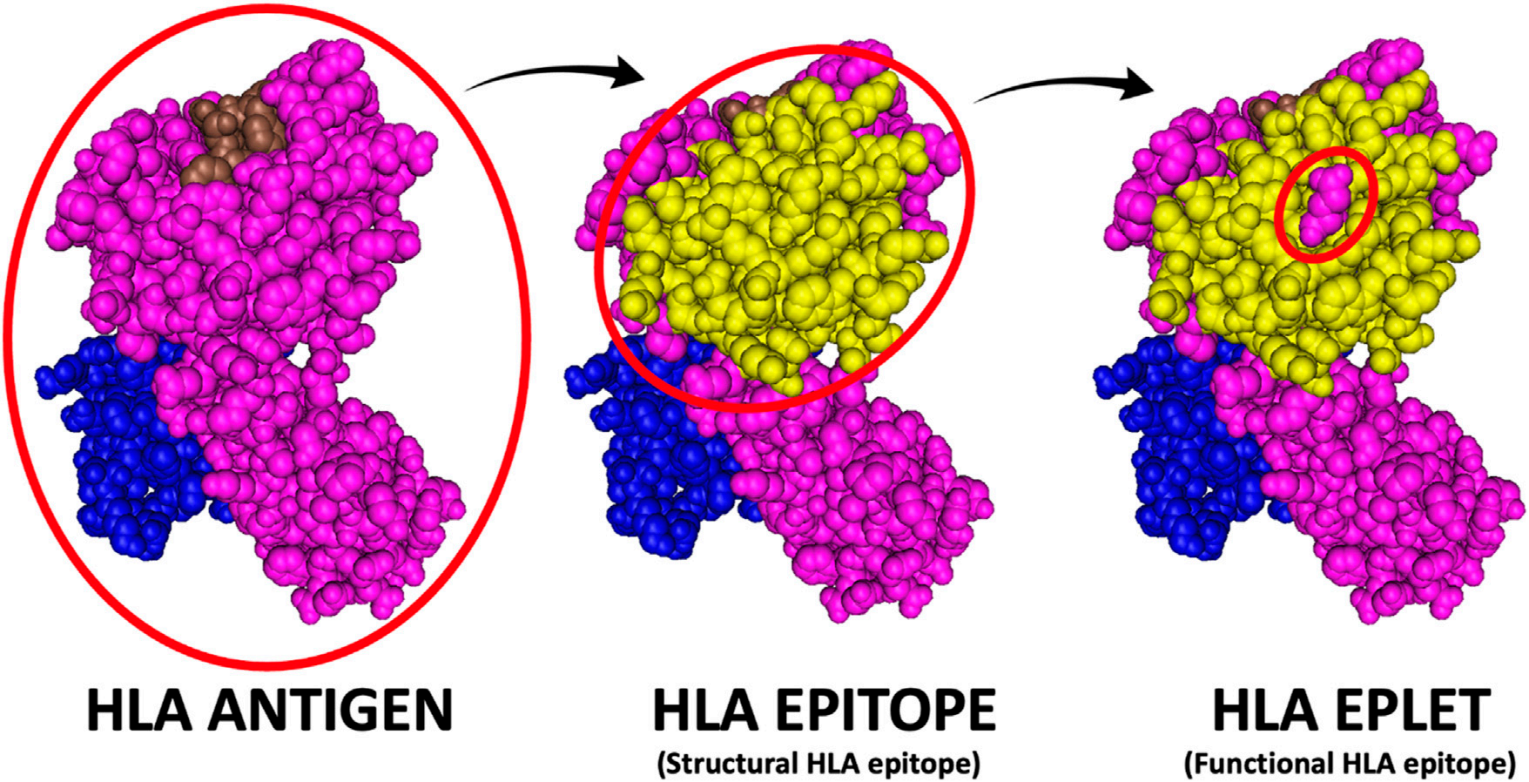
HLA antigen versus epitope versus eplet. The HLA antigen represents the entire immunogenic portion of the HLA molecule, while the HLA epitope (or structural epitope) represents the surface structure comprising up to 25 amino acids residues that serve as target for antibody recognition and anchor for the antibody binding and the HLA eplet represents patch of one or few amino-acid residues constituting the functional main target of antibody binding (i.e., the functional epitope).

It is well established that HLA antibodies do not bind to the entire HLA antigen but to distinct areas in the antigen’s surface called HLA epitopes (Duquesnoy, 2001; Duquesnoy et al., 2004; Silva et al., 2010). The epitope is directly contacted by the antibody hyper-variable regions (paratope). The structural epitope is a large surface structure comprising up to 25 amino acids residues that serve as target for antibody recognition and anchor for the antibody binding (Figure 5). Central to the structural epitope is a patch of one or few surface-exposed amino-acid residues constituting the functional epitope (Tambur and Das, 2023). The functional epitope (HLA eplet) comprises at least one polymorphic residue and is an essential component to the antibody binding. Additionally, polymorphic and non-polymorphic amino-acid residues at 15Å distance from an eplet may constitute essential secondary contact points for the antibody (Duquesnoy, 2014).

Therefore, each HLA antigen cannot be simply viewed as a mono-antigenic entity but rather as a collection of multiple potentially immunogenic eplets. HLA eplets can be private, if restricted to one or few HLA antigens, or public if shared by multiple HLA antigens. Public epitopes, which are responsible for cross reactive epitope groups (CREGs), result in cross-reactions among different HLA antigens (Crowe, 2003). Public epitopes can cause the development of broad HLA allo-sensitizations even following exposure to a single mismatched HLA antigen. Within a given CREG, HLA antigens share most of their amino-acid sequence and can differ by one or few mismatched HLA eplets (Duquesnoy, 2016). Conversely, private HLA eplets/epitopes can lead to the generation of monospecific antibodies. A patient’s HLA antigens represent the self-repertoire of eplets against which no antibodies are normally developed. Following transplant, a donor’s mismatched HLA antigens represent the non-self eplet repertoire against which both cellular and humoral immune responses can be developed.

**FIGURE 6:**
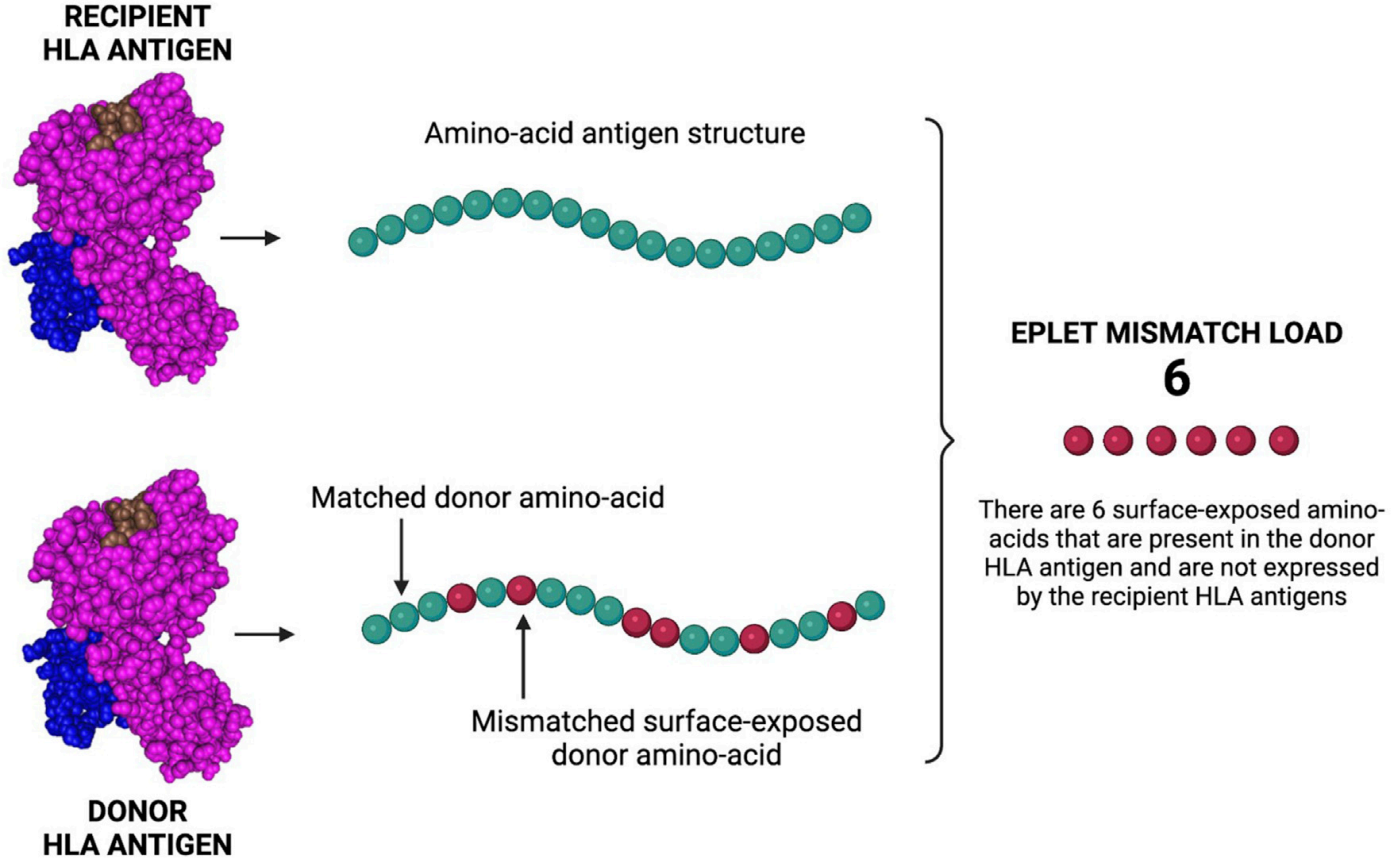
Eplet mismatch load calculation. The Eplet mismatch Calculator enumerates and lists the surface-exposed amino-acids uniquely expressed by the donor HLA antigens. By comparing the molecular (amino-acid) structure of patient/donor HLA antigens, the calculator can identify mismatched amino-acids that can be responsible for the generation of HLA antibodies and AMR. Created with BioRender.com.

In 2001, Duquesnoy developed HLAMatchmaker Duquesnoy (2021). HLAMatchmaker is a computer-based algorithm that defines patient-donor HLA molecular compatibility by determining the amount of mismatched HLA eplets. By comparing the amino-acid structure of the HLA antigens of patient and donor, the calculator can identify surface-exposed amino-acids that are present in the donor-derived HLA antigens and absent in the recipient HLA antigens (Figure 6).

The introduction of HLAMatchmaker created a new paradigm for HLA compatibility and provided a valuable alternative to the conventional HLA antigen match. HLAMatchmaker is able to identify donor-recipient pairs that, although antigen-mismatched by conventional matching algorithm, can be considered highly compatible by virtue of being very similar at the molecular (eplet) level. Essentially, this matching algorithm can significantly increase the pool of compatible donors, especially for a broadly sensitized patient (Duquesnoy et al., 2004). HLAMatchmaker, and its successor calculator in the HLA Eplet Registry (https://www.epregistry.com.br/calculator/index.html) have been extensively used to determine the impact that the amount of mismatched HLA eplets have in the short and long-term allograft survival (Silva et al., 2010; Sullivan et al., 2015; Stapleton et al., 2020; Ladowski et al., 2021; Willicombe et al., 2018; Meneghini et al., 2018). In virtually every organ, adult and pediatric setting, deceased and living donors, the higher the amount of molecular match, the less likely it is for the patient to experience generation of dnDSAs and allograft rejection. Although just a small fraction of HLA eplets have been verified, and the immunogenicity of each eplet must still be verified, HLA eplets represent a powerful biomarker for dnDSA generation, AMR, and allograft rejection.

### 3.3 Other molecular mismatch approaches

Although the primary focus of this manuscript is HLA eplet mismatch and PIRCHE-II, it is important to mention that several other molecular mismatch algorithms have been developed which have been proven to correlate with clinical outcomes.

The Electrostatic Mismatch Score (EMS)-3D was developed to capture and compare the physiochemical disparity between donor and recipient HLA alleles (Mallon et al., 2018). Several studies support associations between EMS-3D and certain clinical outcomes. EMS-3D has shown strong associations with dnDSA formation across Class I and Class II loci (Mallon et al., 2018). In a cohort of 654 adult and pediatric renal transplants, Wiebe et al. (2018) found that the electrostatic model closely correlated with HLA eplet mismatch, thus demonstrating that both algorithms are equally good at predicting primary allograft rejection.

The HLA Epitope MisMatch Algorithm (HLA-EMMA) was developed to predict and enumerate the number of donor-derived mismatched solvent-accessible amino-accessible amino-acids. Solvent accessibility is an important characteristic for interaction with immunoglobulin (https://hla-emma.com/) (Kramer et al., 2020). A few studies support the association between HLA-EMMA scores and some clinical outcomes. A study by Santos et al. recently found that HLA-EMMA has similar associations as eplet mismatch and PIRCHE-II to dnDSA formation (Santos et al., 2024). A different study of 274 kidney transplant recipients by Ellison et al. (2023) found that HLA-EMMA had an association with dnDSA formation and AMR risk, with improved predictive power when combined with PIRCHE-II.

## 4 Molecular matching and its role in transplantation

### 4.1 Molecular matching to increase the donor pool for sensitized patients

As discussed, one challenge in organ transplantation is difficulty finding compatible donors for highly sensitized patients, leading to these patients facing prolonged waiting times and accumulating on the waitlist (Heidt et al., 2019). Traditional HLA matching unnecessarily excludes compatible organs from appearing as potential matches due to the limited granularity of serologic antigen typing. Several studies have validated the role of eplet matching over conventional HLA antigen matching in solid organ transplantation to identify compatible donor-recipient pairs. As early as 1969, it was recognized that highly sensitized thrombocytopenic patients benefit from platelets infusions from donors matched at the CREG-level (Yankee et al., 1969). Despite the HLA antigen mismatch, platelets donors expressing HLA antigens within the patients CREG groups are more likely to recover their platelet counts as compared to CREG-mismatched donors. Duquesnoy and Askar (2007) retrospectively analyzed a cohort of 21,270 HLA-DR antigen matched kidney transplants that, by conventional antigen matching, were mismatched at their HLA-A and -B antigen but compatible by HLAMatchmaker. As compared to the graft survival of the zero HLA-A and–B antigen mismatched cases, they found that cases with 0–2 mismatched eplets (classified by contiguous amino acid triplets at that time) performed similarly. This finding supported the idea that Class I epitope matching with HLAMatchmaker could increase the pool of compatible donors for sensitized patients. In a later paper, Duquesnoy et al. (2004) expanded on this work to demonstrate how in patients with an average cPRA >96%, HLAMatchmaker was able to identify compatible donors in 92% of cases. As briefly mentioned, the Eurotransplant Acceptable Mismatch program was created with the intent of harvesting eplet mismatch information to increase the pool of acceptable organs for highly sensitized patients (Heidt et al., 2019). This program aims to identify donors that, despite their mismatched HLA antigens, are predicted to have a negative flow crossmatch and thus a lower immunological risk. For highly sensitized patients, organ allocation based on eplet mismatch analysis, and the identification of acceptable antigens appears to have rejection rates comparable to non-sensitized patients (Heidt et al., 2019). In another study, Tafulo et al. (2021a) were able to demonstrate that an eplet-based PRA was significantly lower than traditional cPRAs, which would greatly increase the odds of transplant in many highly-sensitized patients.

Importantly, in a decision analytical modeling study, Nguyen et al. demonstrated that inclusion of an eplet mismatch-based acceptable mismatch program in the Australian kidney allocation model would improve transplantation rates and waiting times for highly sensitized waitlist patients, with a net benefit to the overall system in terms of quality-adjusted life years and patient costs (Nguyen et al., 2014). All these data demonstrate the usefulness of determining the level of compatibility beyond the HLA antigen and at a molecular level.

While PIRCHE-II is helpful for risk-stratifying for T cell rejection, dnDSA, and AMR, it is unable to provide additional information on potential donor compatibility for pre-existing DSA. However, the Tmem (T-cell memory) module of PIRCHE can help identifying HLA antigens that express peptides that were present in the HLA antigen of the previous donor and therefore represent repeat mismatches (Tomosugi et al., 2021; Peereboom et al., 2021).

### 4.2 Molecular matching to improve long-term outcomes and reduce immunosuppression

As discussed, modest increases in long-term graft survival have been one of the recalcitrant issues in transplantation, contributing to the significant re-transplantation rate observed, especially for kidney transplant and pediatric recipients. There is a growing body of evidence that optimized molecular matching reduces the risk of graft rejection and failure, increasing long-term survival. Furthermore, better matched donor-recipient pairs appear to allow for the recipient to achieve better long-term graft survival while requiring lower levels of immunosuppression, potentially decreasing the risk of opportunistic infections, post-transplant malignancies, and other adverse effects.

Eplet-based molecular matching has been shown to correlate with dnDSA risk, especially based on HLA Class II mismatches. (Key articles related to this section are summarized in Table 3.) In a retrospective cohort of 146 sensitized renal transplant patients, Dankers et al. (2004) described a strong correlation between the number of mismatched HLA Class I triplets (i.e., eplets) and the proportion of patients developing post-transplant dnDSA. Compared to patients with zero mismatched triplets who did not develop dnDSA, 94% of patients with 11–12 mismatched triplets developed post-transplant dnDSA. They concluded that eplet-matching donor-recipient pairs would reduce the incidence of acute and chronic AMR and promote long-term allograft survival. Given the proportion of transplant patients developing Class II dnDSAs and the correlation between the amount of Class II mismatches and graft survival, Duquesnoy et al. investigated the relationship between HLA class II eplet mismatch and the likelihood of developing Class II antibodies. In a cohort of 75 sensitized solid organ transplant recipients who were being considered for re-transplantation, the probability of developing Class II dnDSAs increased with an increasing number of eplet mismatches at HLA-DR, -DQ, and -DP (Duquesnoy et al., 2008a). Haririan et al. subsequently used HLAMatchmaker to investigate the effect of Class II eplet mismatch on allograft survival. In a cohort of 76 patients with HLA-DR, -DQ conventional antigen typing, the degree of HLA-DQ eplet mismatch was a significant independent predictor of allograft failure (Haririan et al., 2006). Similar correlations were seen for the degree of total HLA-DR, -DQ eplet mismatch, as well as for total Class I and II eplet mismatch. Eventually, it became evident that the mismatched eplet load, especially at HLA-DR and–DQ, played a significant role in the development of dnDSAs and allograft rejection.

**TABLE 3:** Summary of key studies examining molecular mismatch approaches and post-transplant outcomes.

Study	Cohort	Donor type	Type of HLA matching/Comparison	Outcome	Summary
Dankers et al. (2004)	146 sensitized kidney transplant patients	Deceased	Triplet – Class I	dnDSA	Correlation between number of Class I triplet mismatches and production of dnDSA against rejected donor organ (*p* < 0.0001, r^2^ = 0.99)
Duquesnoy et al. (2004)	75 sensitized solid organ transplant recipients	Mixed	Triplet- Class II	dnDSA	Sensitized patients with Class II dnDSAs had higher counts of eplet mismatches at HLA-DR (*p* < 0.0001), -DQ (*p* < 0.0001), and -DP.
Haririan et al. (2006)	76 kidney transplant recipients with HLA-DR, -DQ conventional antigen typing	Mixed	Triplet – Class I and II	Graft Failure	Degree of HLA-DQ eplet mismatch, total -DR and -DQ Class II eplet mismatch, and total eplet mismatch were significant predictors of allograft failure (*p* = 0.029, *p* = 0.045, and *p* = 0.034 respectively)
Wiebe et al. (2013)	45 transplant recipients who developed Class II dnDSA	Mixed	Eplet – Class II	dnDSA	Eplet mismatch load at HLA-DR (p < 0.02) and -DQ (*p* < 0.001) outperformed high-resolution whole antigen mismatch as a predictor of dnDSA.
Wiebe et al. (2017)	596 kidney transplant patients with serial tacrolimus monitoring	Mixed	Eplet – Class II	dnDSA	High alloimmune risk patients (with >11 HLA-DR and -DQ mismatched eplets) were more likely to develop dnDSA and had a higher proportion of sub-therapeutic tacrolimus trough levels than high alloimmune risk patients who did not develop dnDSA.
Wiebe et al. (2019)	664 kidney transplant recipients	Mixed	Eplet – Class II	TCMR, AMR, dnDSA	Retrospectively identified eplet-based Class II alloimmune risk categories that correlated with Banff ≥1A T cell–mediated rejection (*p* < 0.0001), HLA‐DR and -DQ dnDSA (*p* < 0.0001), and antibody‐mediated rejection (*p* < 0.0001)
Davis et al. (2021)	444 kidney and kidney/pancreas recipients	Mixed	Eplet – Class II	dnDSA	Patients with Class II alloimmune risk and a mean tacrolimus <6 ng/mL versus >8 ng/mL had increased risk of HLA-DR and -DQ dnDSA at 1 year post-transplant (*p* = 0.04)
Meneghini et al. (2018)	330 kidney transplant recipients	Living	Eplet – Class II	AMR	Higher degree of Class II eplet mismatch was associated with higher risk of AMR amongst recipients with baseline negative crossmatches (*p* = 0.019)
Tafulo et al. (2021b)	151 kidney transplant recipients	Living	Eplet – Class II	AMR	High Class II eplet mismatch was an independent predictor of AMR and a better predictor of AMR than antigen mismatch (*p* = 0.023)
Philogene et al. (2020)	110 pediatric kidney transplant recipients	Mixed	Eplet – Class I and II	dnDSA, AMR, Graft Failure	Degree of Class II eplet mismatch significantly increased the risk of dnDSA development (*p* < 0.001). An association was not observed for rejection or graft loss in this small population, nor for Class I eplets
Sypeck et al. (2020)	196 pediatric recipients of primary renal allografts	Mixed	Eplet – Class I and II	dnDSA, Graft Survival	HLA class I eplet mismatch was a significant predictor of graft survival (HR 1.05 per mismatch, 95% CI: 1.01–1.09). HLA class II eplet mismatch was a significant predictor of retransplant (HR 0.98 per mismatch, 95% CI: 0.97–1.00). Class I eplet mismatches were also associated with Class I dsDSA development, while Class II eplet mismatches were associated with Class II dnDSA.
Lachmann et al. (2017)	2,787 kidney transplant recipients	Mixed	PIRCHE-II	dnDSA, Graft Survival	PIRCHE-II independently risk-stratified dnDSA formation (*p* < 0.001) and graft survival (*p* = 0.021)
Geneugelijk et al. (2018)	2,918 kidney transplant recipients	Mixed	PIRCHE-II	Graft Failure	Logarithmic PIRCHE-II score was an independent predictor of graft failure (*p* = 0.003)
Senev et al. (2022)	893 kidney transplant recipients	Mixed	PIRCHE-II	TCMR	Certain Class II PIRCHE-II scores for were independent predictors of TCMR and graft failure; Class I PIRCHE-II scores were not associated with either TCMR or graft failure

AMR, antibody mediated rejection; dnDSA, *de novo* donor specific antibodies; HLA, human leukocyte antigen; TCMR, T cell mediated rejection.

More recently, Wiebe and colleagues investigated the role of HLA-DR and -DQ eplet mismatch load as a potential biomarker for allo-immune risk. In a cohort of 45 transplant recipients who developed Class II dnDSA (HLA-DR, HLA-DQ, or both) they found that the HLA eplet mismatch load outperforms whole antigen mismatch as a predictor of dnDSA (Wiebe et al., 2013). When looking at the eplet mismatch load that consistently resulted in the development of dnDSAs, they found that ≥10 HLA-DR and ≥17 HLA-DQ eplet mismatches strongly correlated with the development of locus-specific dnDSA. The authors concluded that -DR, -DQ eplet matching has the potential of being used as a biomarker for the risk of developing Class II dnDSAs and to improve long-term allograft survival. In a subsequent retrospective study of 596 single-center kidney transplant patients with 50,011 serial tacrolimus trough levels, Wiebe and colleagues demonstrated that, in patient with sub-therapeutic tacrolimus through levels, the eplet mismatch load at HLA-DR and–DQ strongly correlated with the development of dnDSA (Wiebe et al., 2017). High alloimmune risk patients (>11 HLA-DR and–DQ mismatched eplet) who developed dnDSA had a higher proportion of sub-therapeutic tacrolimus trough levels. However, low alloimmune risk patients (≤11 HLA-DR or–DQ mismatched eplets) tolerated the same percentage of sub-therapeutic tacrolimus through levels without developing dnDSAs (although it is important to note that the inclusion of -DR and -DQ allelic matched pairs in this analysis—who are unable to form dnDSA targeted against those loci—may have led to underestimation of dnDSA risk in the low alloimmune risk group). In 2019, Wiebe and colleagues published another retrospective study on 664 kidney transplant cases where the single molecule HLA-DR and–DQ eplet mismatch load was correlated with serologic, histologic, and clinical outcomes (Wiebe et al., 2019). In this study cohort, the authors calculated the individual molecular mismatch load at HLA-DRB1, -DRB3/4/5 and -DQA1/DQB1 and correlated this result with the development of dnDSA, AMR and ≥Banff-1A cellular rejection. They demonstrated the existence of three alloimmune risk categories: low, intermediate, and high. The observations from Wiebe and colleagues were confirmed with a 444 patient-donor validation cohort, thus solidifying the role of HLA-DR and–DQ eplet mismatch load as a strong predictive biomarker for the development of dnDSA and allograft rejection (Davis et al., 2021). Moreover, early data in liver transplant recipients suggesting that eplet mismatch load at HLA-DQB1 may be associated with risk of T-cell mediated rejection (TCMR) (Ono et al., 2021).

These findings have also been recapitulated in living donor cohorts. Meneghini et al. (2018) reviewed a cohort of 330 living donor kidney transplants who were matched by conventional HLA antigen methods and found that patients who had a higher degree of eplet mismatch were at higher risk of developing AMR. In a cohort of 151 living donor kidney transplants, Tafulo and colleagues found that high HLA eplet mismatch was an independent predictor of AMR while HLA antigen mismatch was not (Tafulo et al., 2021b). HLA eplet mismatch load was also significantly better predictor of AMR than HLA antigen mismatch. They too concluded that eplet matching in living donor kidney transplant leads to successful donor-recipient pairing and is a potential biomarker for personalized assessment of alloimmune risk.

Eplet-based molecular matching also provides an advantage to pediatric populations. Pediatric patients face the challenges of long-term allograft loss due to chronic-AMR (cAMR) and increasing sensitization with recurrent transplantation (Sun and Yang, 2013). Optimizing donor-recipient HLA compatibility in these transplants is critical to preventing the formation of dnDSA, avoiding sensitization, and minimizing the risk of cAMR. Philogene et al. (2020) retrospectively analyzed eplet mismatches and allograft outcomes in a cohort of 110 pediatric transplant patients undergoing primary kidney transplant and with a mean follow up of 5.8 years. They found that the risk of dnDSA development was significantly increased with greater degrees of eplet mismatch. However, there was no association between degree of eplet mismatch and graft loss. Starting in 2011, the Transplantation Society of Australia and New Zealand introduced a point-bonus system into their national allocation algorithm for any child under the age of 18 years who had been on dialysis for more than 1 year. To increase the benefit of this new allocation system, Kausman et al., (2016) utilized HLA eplet matching to minimize the risk of allo-sensitization and improve time to transplantation. In a cohort of 19 low Class II eplet mismatch kidney transplants followed for 12 months they reported low rates of dnDSAs in the first year of transplant. To address the question of long-term allograft eplet matching in pediatric patients, Sypek et al. (2020) evaluated 196 pediatric recipients of primary renal allografts from an Australian Registry. Their study showed that HLA class I eplet mismatch was a significant predictor of graft failure. HLA eplet mismatch was associated with increased likelihood of re-transplantation and class-specific eplet mismatch was a strong predictor of dnDSA formation. While long term data regarding transplant outcomes and HLA eplet matching is limited in this patient population, the lower incidence of dnDSA in low eplet mismatch pairs is compelling enough that several centers have already integrated eplet matching with HLAMatchmaker to their clinical practice. Pediatric patients also struggle with medication non-adherence. The strong correlation between graft loss rates in non-adherent recipients with of the eplet mismatch load at HLA-DR and HLA-DQ provides an opportunity to properly guide and reinforce adherence to patients who are at an increased risk of rejection (Philogene et al., 2020).

Indirect alloreactivity measured using PIRCHE-II also has a growing body of evidence supporting its association with dnDSA formation and long-term graft outcomes. In one of the first analyses of PIRCHE-II, Otten et al. retrospectively analyzed 21 donor-recipient pairs in which the recipient experienced graft failure (Otten et al., 2013). Of the 49 HLA Class I mismatches present in the 21 pairs, dnDSAs were present for 38 of the mismatches, which had significantly more PIRCHE-II mismatches than the non-immunogenic HLA mismatches. Notably, PIRCHE-II locations poorly overlapped with eplet mismatches, with 62% of HLA Class I-derived polymorphic residues being potentially alloreactive as PIRCHE-II mismatches but not as eplet mismatches. Although a small cohort, these findings were recapitulated in a large restrospective cohort analysis by Lachmann et al. (2017) including 2,787 kidney transplant recipients in Germany. In this study, both PIRCHE-II and eplet mismatches were correlated with dnDSA formation, with PIRCHE-II an independent predictor of dnDSA formation in multivariate analysis controlling for eplet mismatches (Lachmann et al., 2017). Interestingly, the probability of dnDSA formation was increased primarily for HLA-DRB1 and -DQ mismatches, with Class I mismatches having a lower likelihood of dnDSA formation. Importantly, PIRCHE-II was also predictive of allograft survival. Interestingly, Lachmann et al. did find some correlation between PIRCHE-II score and eplet mismatches, but each scoring system independently predicted graft survival and dnDSA incidence. In an important parallel study, Geneugelijk et al. (2018) retrospectively examined 2,918 kidney transplant recipients. PIRCHE-II score was found to predict early and late graft failure. PIRCHE-II as a predictor of dnDSA formation has also been demonstrated in liver and heart transplant patients (Mangiola et al., 2022; Ono et al., 2021; Hamada et al., 2020). Furthermore, there is a growing body of evidence that because of the cooperation between T and B cell in the generation of a primary allograft rejection, T cell activation and T cell memory play a significant role in this mechanism, and combining T cell and B cell mismatch prediction scores improves risk stratification for dnDSA formation and AMR (Mangiola et al., 2022; Ellison et al., 2023).

PIRCHE-II also has a growing body of evidence demonstrating its correlation with better long-term outcomes in transplant recipients on reduced or calcineurin inhibitor (CNI) sparing immunosuppression regimens. In a retrospective study of 41 liver transplant recipients on CNI-free immunosuppression by Meszaros et al. (2020), PIRCHE-II scores were significantly higher in patients who had rejection episodes, and PIRCHE-II was an independent predictor for graft survival. In a retrospective analysis of kidney transplant recipients who underwent maintenance immunosuppression reduction due to COVID-19 infection, Castrezana-Lopez et al. found that PIRCHE-II scores and specifically HLA-DR PIRCHE-II were predictive of dnDSA formation (Castrezana-Lopez et al., 2023). PIRCHE-II scores have also been associated with increased risk of TCMR specifically. In a retrospective study by Senev et al. examining 893 kidney transplant recipients, incremental increases in PIRCHE-II score were associated with TCMR risk (p = 0.009), with this relationship driven entirely by HLA Class II scores (Senev et al., 2022). Importantly, PIRCHE-II scores for HLA-DRB1 and DQB1 in this same study were associated with all-cause and death-censored graft failure (p = 0.03 and p = 0.02, respectively). Another study by Betjes et al. (2022) found a similar correlation between PIRCHE-II scores and vascular TCMR. An association between PIRCHE-II score and TCMR has also been demonstrated in liver transplant recipients (Ono et al., 2021).

There are however several challenges associated with molecular match approaches currently. The most significant challenge is a technological one: true high-resolution HLA genotyping with next-generation sequencing is not universally available in clinical settings, especially at the speeds that would be required for use in some applications (such as in deceased donor allocation) (Lemieux et al., 2021). While nanopore and rapid throughput next-generation sequencing approaches have been described and are starting to become commercially available, their use is currently limited (De Santis et al., 2020; Liu et al., 2021). More readily available technologies, including sequence-specific primer and sequence-specific oligonucleotide probe typing do not provide true high-resolution typing (Smith et al., 2019). Furthermore, at least for eplet mismatch typing, there are multiple extant algorithms, which are not universally concordant (Tassone et al., 2020). Additionally, eplet mismatches are by their nature theoretical; not all eplet mismatches have been proven as true epitopes that antibodies bind to. While the HLA Eplet Registry has begun to try to address this challenge by tracking antibody verification of eplets, there has been criticism that antibody verification within the registry is not very transparent and some antibody-verified eplets are not associated with immune responses (Tambur and Das, 2023). Currently, eplet mismatch scores do not always discriminate between antibody-verified versus unverified eplets. Furthermore, as antibodies are verified *in vitro*, technique-dependent variations of *in vitro* reactivity present a challenge for antibody verification (Duquesnoy, 2014). Regarding PIRCHE-II specifically, this algorithm is relatively new compared to eplet mismatch analysis, and as a result has a less robust body of evidence, including some studies that do not show a meaningful association with rejection rates (Kok et al., 2022). Furthermore, direct comparisons of eplet mismatches and PIRCHE-II and the combination of the two are lacking, as are prospective studies. While all these challenges can be overcome, further development in all these areas will be required as molecular match approaches advance.

## 5 Immunological responses to non-classical HLA genes and to non-HLA genes

As we have just reviewed, the vast majority of studies have correlated graft failure with genetic differences at the classical HLA genes (HLA-A, -B, –DR, -DQ) or with immunological response against the antigenic product of classical HLA genes. A small number of studies have examined additional HLA genes, such as HLA-C, and HLA-DP. HLAMatchmaker and the HLA Eplet Registry were also expanded to include the major-histocompatibility-complex (MHC) Class I–related chain A (MICA), enabling its inclusion in compatibility analyses (Duquesnoy et al., 2008b). While immune responses against foreign tissues and organs is more commonly due to antigenic differences at the level of the major histocompatibility complex, genetic differences outside the classical MHC gene region also play a role in alloimmune response and can generate potentially incompatible antigens. It is for this reason that HLA-identical siblings who undergo solid organ and stem cell transplantation can still have graft failure and graft-versus-host disease (GvHD) (Simpson, 1991; Dierselhuis and Goulmy, 2009). While classical HLA genes represent the single largest source of immunologic match (and importantly, the largest immunologic target for preformed DSAs), non-classical HLA genes, minor histocompatibility antigens, and non-HLA genes cumulatively comprise a large portion of immunologic match. With the advent of clinically available sequencing techniques, understanding the role of these genes represents a significant opportunity to potentially improve long-term allograft survival.

Several specific minor Histocompatibility Antigens (mHAs) have been identified. In female patients who received kidney allografts from male donors, worse survival outcomes were observed when compared with all other gender combinations (Gratwohl et al., 2008). This correlation between male-to-female transplant pairs and acute graft rejection has been partly attributed to the Y-chromosome male-enhanced antigen (MEA1) gene and the generation of H-Y antibodies (Tan et al., 2008). Technical advances in genomics allowed us to determine that recipient-donor differences in mHAs also play an important role in host alloimmune responses (Simpson, 1991). Because the genes that encode mHAs are scattered throughout the Human genome, the sources of potential mHA–mismatches between recipients and donors is often significant, even between HLA-matched siblings (Dierselhuis and Goulmy, 2009).

Since the initial draft sequences of the first human genomes over 20 years ago, genome-wide SNP maps have been generated across the major human populations facilitating genome-wide genotyping panels typically including >5,00,000 to several million SNPs. Subsequent advances in sequencing technologies facilitated whole exome sequencing (WES) and whole-genome sequencing (WGS). WGS of large reference populations such as the 1,000 Genome Project (1KGP) has facilitated characterization of common and rare genetic variants (Abecasis et al., 2010). 1KGP and similar studies have shown that, on average, two unrelated humans differ across their genomes by ∼3.5 million to 10 million polymorphisms, depending on ancestry. The International Genetics & Translational Research in Transplantation Network (iGeneTRAiN) has created and aggregated the genome-wide datasets of >56,000 recipient-only or donor-recipient pairs to perform large scale mHA and HLA studies (Keating et al., 2015). The importance of genome-wide mHAs has been demonstrated even after controlling for HLA mismatch. Genome-wide mismatch analyses of transmembrane and secreted proteins in 477 prospectively collected kidney transplant pairs from an iGeneTRAiN study revealed that mismatches at non-HLA genes independently associate with graft loss in a multivariable model adjusted for HLA eplet mismatch and serotype (Reindl-Schwaighofer et al., 2019).

Single-gene mHAs impacting graft survival have also been identified using genome-wide approaches. A recent iGeneTRAiN study performed on a large kidney transplant cohort showed that a single SNP in LIMS1 is associated with increased risk of acute rejection (Steers et al., 2019). The LIMS1 locus (also a copy-number variant locus) encodes an mHA that can be expressed on the cell surface and is therefore a potential endothelial cell antigen. Interestingly, recipients that were homozygous for the LIMS1-variant rs893403, had significantly reduced mRNA levels (Steers et al., 2019). Kidney patients transplanted with donors expressing a LIMS1 in “genomic collision” with the recipient LIMS1 variant (where the recipient is variant-homozygous and the donor is variant-heterozygous) demonstrated a 58% increase in the risk of developing acute rejection (combined HR, 1.63; 95% CI, 1.37–1.95; p = 4.7 × 10^−8^), with confirmation of the presence of LIMS1-specific antibodies, thus indirectly proving the existence of an alloimmune response toward a mismatched non-HLA antigen (Reindl-Schwaighofer and Oberbauer, 2019).

The association between the genetic LIMS1 variants and the risk of developing acute kidney allograft rejection brings up another important concept: copy-number variations (CNV). Genomic CNVs are structural gene variants that are the result of deletion and/or duplication of a DNA segment (Hastings et al., 2009). Due to CNVs, genes can gain or lose functions and their encoded protein can be over expressed, under-expressed, or lacking altogether. The LIMS1 rs894303 variant is a great example of mutation-induced loss-of-function (LoF). For patients expressing the LIMS1-variant, the inherent reduction in the endothelial expression of LIM protein translates into the recognition of LIM as a non-self protein, and to the development of an immune response against a non-HLA antigen. LoF mutations can be due to insertion or deletions, CNVs, or single nucleotide variants (SNVs). SNVs have a frequency of <1% in the population and can disrupt a specific part(s) of a gene or the function of the entire gene. When LoF affects genes coding functional proteins, the affected protein in the donor organ may act as neo-antigen and induce alloreactivity. In a systematic survey of LoF variants in the human genome, MacArthur et al. (2012) investigated the genome of 185 humans to identify rare and common LoF variants. They estimated that, on average, the human genome contains ∼100 genuine LoF variants, with ∼20 genes having LoFs in both copies. By investigating more than 15,500 human protein-coding genes in over 2,000 individuals of diverse ancestry, more than 5,00,000 SNVs were identified. On average, each individual can express more than 13,000 SNVs, with about 2% predicted to impact the function of more than 300 genes (MacArthur et al., 2012; Tennessen et al., 2012).

Whole exome studies indicate that the human genome is enriched in mutations in “disposable” or redundant genes as compared to genes that are essential for basic functional molecular processes. These data suggest that there are numerous mutations that occur at an individual level (“personal” genome) (Lim et al., 2013) and represent plausible sources of non-HLA incompatibility and non-HLA graft rejection. In conjunction with polymorphisms in the classical HLA genes, precise determination of the “personal” genomic makeup can be the foundation of personalized transplant medicine.

## 6 Discussion: beyond traditional HLA matching: what is ready for primetime?

Thus far, we have explored the mechanistic underpinnings of molecular matching and non-HLA genetics in organ allocation and post-transplant personalized medicine, as well as the existing clinical evidence in these areas. In this section, we will discuss potential practical applications of these technologies. There are certain applications that we believe are “ready for primetime” (i.e., ready for at least initial implementation within systems like UNOS in the US in the next 5 years), while others we believe are promising but require further development, refinement, or exploration of potential negative externalities. Although we focus on the UNOS system in this discussion as it is the largest organ sharing system in the world by an order of magnitude, many of the applications we discuss would similarly provide benefit in smaller national and multinational systems (and in fact, may be easier to implement in these smaller systems in some cases). This section represents our assessment of what the near future of organ allocation and post-transplant personalized treatment should—and may—look like with regards to implementing non-traditional molecular HLA and non-HLA approaches.

### 6.1 Not yet ready for primetime: molecular matching in deceased donor organ allocation

Many studies on molecular match (eplet and PIRCHE-II) have been performed on retrospective cohorts of patients transplanted with cadaveric organs. Nevertheless, the question remains as to whether and how molecular HLA matching can be applied to deceased donor transplantation. Molecular match analysis would likely produce improved long-term graft outcomes if it replaced traditional allelic HLA matching in organ allocation, as indicated by the large number of publications that have highlighted the clinical usefulness of molecular match analysis in determining the long-term allograft outcome in solid organ transplantation. There are, however, a few issues that need to be addressed, including technical, logistical, and conceptual barriers that still prevent the application of this matching strategy in deceased donor allocation. On the technical and logistical front, rapid high-resolution genotyping at the speed and scale required for HLA matching with cadaveric donors is not currently available in most clinical settings around the world, especially in the US (Lemieux et al., 2021). Rapid high-resolution HLA sequencing using nanopore approaches has been reported (Liu et al., 2021), as has rapid near-high-resolution HLA typing using next-generation sequencing platforms (De Santis et al., 2020). However, these approaches are not in widespread use and are still costly and labor intensive. Moreover, none of the deceased donor sharing systems have the full informatics infrastructure available to accept any HLA allele and to perform match runs at the molecular level. Additionally, rare and null HLA alleles are currently not included in the sharing systems databases and may require analysis outside the current workflow (Lemieux et al., 2021). As a result, no allocation system currently includes molecular matching analysis in allocation algorithms. These hurdles are, however, surmountable, as evidenced by the Eurotransplant program’s handling of intermediate resolution typing (Eurotransplant, 2024). Importantly, these challenges scale with geographical complexity and throughput requirements, meaning implementation in smaller national systems with a small number of HLA labs would be within easier grasp than implementation in a very large system like the UNOS system in the US.

Moreover, prospective studies must be done to determine the usefulness and full implications of including molecular match as one of the parameters used to allocate organs. Changes to deceased donor allocation models are complex, legalistic tasks with widespread implications for all stakeholders, including regarding equity and fairness of allocation. Specific questions need to be answered before molecular matching can be implemented at the allocation level. For example, while molecular match approaches represent the degree of match continuously based on the number of eplet mismatches or number of potential PIRCHE-IIs, the alloractive risk of increasing mismatch is decidedly non-linear. In fact, much of the analysis of PIRCHE-II scoring relies on logarithmic transformation of the scores (Geneugelijk et al., 2018). And while “high risk” cutoffs for some eplet mismatch numbers have been established, especially for HLA-DR and -DQ eplet mismatch counts, data are relatively limited. Large dataset analyses will be required to determine high-validity high-risk cutoffs for molecular matches and accurate continuous scoring systems. Alignment of eplet matching software into a single consistent system will also be an important intermediate step (Tassone et al., 2020). Even once continuous scoring systems are established, understanding how to weight molecular match analysis within the broader context of deceased donor allocation presents its own considerable challenge. Preliminary modeling studies have been performed by Niemann et al. (2021). exploring the incorporation of eplet mismatch for unacceptable antigens and PIRCHE-II scores for allocation in the Eurotransplant kidney allocation system. In this simulation, substitution of HLA match grade for PIRCHE-II based allocation led to lower average PIRCHE-II scores with comparable waiting times with the current allocation model, although the number of HLA allelic mismatches increased slightly. It is important to note that HLA eplet mismatch was not incorporated in the modeled allocation algorithm (only PIRCHE-II scores were), which may not represent optimal risk assessment (Ellison et al., 2023), and likely explains the increase in HLA mismatches observed. Further predictive modeling studies of potential inclusion of molecular match would be required to ensure there are no untoward effects that would compromise the equity or fairness of allocation, as would considerable discussion with the many stakeholders involved in deceased donor organ allocation. While a promising potential avenue for the future, the use of molecular matching in deceased donor organ allocation will require considerable technological, logistical, and scientific development to make reality, given the degree of scrutiny on and widespread impact of deceased donor allocation.

### 6.2 Informative and potentially ready for primetime: molecular matching for physicians evaluating deceased donor organ offers

While the ability to include molecular match analysis in organ allocation is some years away (as discussed above), it would be possible to start providing physicians evaluating potential organ offers with molecular match scores, when donor and putative recipient high-resolution HLA genotyping are available. Although some similar challenges exist as far as many centers not having access to rapid high-resolution HLA genotyping capabilities, the combination of high-resolution recipient HLA typing and high-resolution donor typing (or when needed, intermediate-resolution typing with imputation—which many more centers have access to Geneugelijk et al. (2017)) would allow for relatively accurate molecular match scores to be provided in the context of some deceased donor organ offers, even if the data cannot be incorporated into allocation. Making these scores and basic interpretations available to physicians evaluating organ offers has the potential to influence organ acceptances and declinations in a way that may improve long-term outcomes. Would a kidney with a longer ischemia time but near-perfect molecular matches potentially move from a decline to an acceptance? Would a seemingly reasonable organ for a high-risk recipient with a disproportionately high PIRCHE-II score move from an acceptance to a decline? A pilot study to understand how clinicians would evaluate and utilize these scores would be extremely helpful to answer some of these questions and would not require high-profile changes to organ allocation. Similarly, building the infrastructure to begin collecting longitudinal data on molecular matching within the UNOS system would be extremely helpful for long-term studies understanding the implications of molecular matching (and ultimately, necessary for the eventual incorporation of molecular matching into UNOS allocation). While some of the same challenges discussed above would still exist, they are much easier to overcome in the context of a program that individual centers can choose to leverage or ignore, that does not necessarily require high-resolution donor typing, and that does not directly impact the allocation algorithm.

### 6.3 Ready for primetime: molecular matching-based targeted immunosuppression reduction and increased surveillance

At this point, especially for eplet-based molecular match analysis, there is a considerable body of evidence suggesting that immunosuppression reduction and/or CNI-minimizing immunosuppression can be safely implemented in many low mismatch organ recipients. There seems to be clinical equipoise to warrant a prospective randomized trial on this very question. Importantly, however, the exact criteria for designating a “low-risk” donor-recipient immunologic dyad requires further exploration. While cutoffs for both HLA-DR and -DQ eplet mismatch counts and PIRCHE-II scores have been suggested, these cutoffs likely do not apply universally; rather other donor-, recipient-, and transplant-specific factors are reasonably likely to moderate the relationship between immunologic match and graft outcomes. For example, grafts with longer cold ischemia time are at greater risk for dnDSA formation and subsequent antibody-mediated rejection, perhaps due to more significant ischemia-reperfusion injury producing a greater antigenic load (Senev et al., 2020; Wiebe et al., 2018). The development of polyfactorial risk scores that incorporate molecular matching are likely to be superior for assessing true rejection and graft failure risk, which may be better able to safely guide immunosuppression and/or CNI minimization.

Conversely, as outlined above, there is considerable evidence that patients with increased eplet mismatch and PIRCHE-II scores are at increased risk for graft rejection and failure. It would be reasonable to consider evaluating increased frequency versus standard post-transplant monitoring in the initial post-transplant period in this higher-risk group, perhaps in a randomized trial. Similar constraints regarding current knowledge of which transplant recipients should be considered “higher risk” apply.

### 6.4 Ready for primetime: molecular matching in living organ donation and kidney paired exchange

In living organ donation, molecular HLA compatibility can significantly improve graft survival and quality of life. However, the most significant obstacle to a full implementation of this matching strategy is the probability of finding a low molecular mismatch in the small pool of donors that the patient’s inner circle represents. With only a 25% chance of matching with a sibling, even a larger pool of related donors may not be enough to find a low molecular mismatch donor. Moreover, for highly sensitized patients, the donor pool is—at best—significantly reduced. To improve the chances of finding a more compatible donor, patients need to consider the benefit of uncoupling from their donor and leveraging the extensive donor pool offered by kidney exchange programs. Because of their large pool of potential donors, Kidney Paired Exchange (KPE) programs are becoming an increasingly attractive option for sensitized patients. And to fully harvest the benefit of molecular HLA compatibility matching for all patients, KPE programs offer the possibility of increasing the pool of compatible donors, reducing the time to transplant and improving graft survival and quality of life.

Kidney paired exchange (KPE) describes the mixing and matching of living donor kidneys to find the most optimal and compatible donor-recipient pairs. KPE is a widely practiced strategy and over the years has developed into programs of donation that involve multiple donor-recipient pairs and can span across state and country (Kher and Jha, 2020). KPE programs effectively increased rates of living donation and improved transplant outcomes. A drawback of KPE however is that highly sensitized patients still tend to accumulate within the candidate pool as they fail to find compatible donors based on conventional matching strategies (Holscher et al., 2018). Ferrari et al. addressed this limitation through a simulation of the Australian Paired Kidney Exchange Program (Ferrari et al., 2017). They found that by using eplet matching for entering compatible and incompatible donor-recipient pairs, the majority of compatible pairs were able to find a suitable match, with some gaining a better immunological profile than with their original donor. It also allowed for an increased number of incompatible donor-recipient pairs to find appropriate immunologic matches. Uncoupling compatible living donor pairs is essential to increase the donor pool for incompatible donors. HLA compatibility is not a binary question, but rather a range of possible compatibilities. Traditionally compatible pairs can be uncoupled to find a more compatible pairing. In turn, uncoupled donors can be the one needle in the haystack that a highly sensitized patient has been waiting for. With a sufficiently sized pool, all recipient groups would on average benefit.

One of the KPE programs that has emerged as the leading organization for improving living donor transplantation is the National Kidney Registry (NKR). The NKR is a non-profit organization that has developed into the preeminent KPE network in the United States (Flechner et al., 2018). It has facilitated over 8,600 transplants in the past 15 years (National Kidney Registry, 2024). The NKR uniquely offers a variety of support services for donors and an innovative voucher program that enables individuals to donate in advance of their paired recipient undergoing transplant. This assures donors that their recipients will receive their transplant at the appropriate time and increases the opportunity for a recipient to find a more optimal or compatible match. These innovations have resulted in a steady stream of compatible and non-compatible donors that is making the NKR the largest living donor pool (Kher and Jha, 2020). A large donor pool is not only critical to facilitate transplants for the immunologically hard-to-match recipients, but it also prevents enrichment of the pool with sensitized donors and recipients who are unlikely to match. Furthermore, the larger the pool, the higher the likelihood of finding a more compatible donor with good molecular match. Recently, the NKR introduced a new matching algorithm based on the level of eplet mismatch at HLA-DR and–DQ (Patient-Recipient Kidney Matching, 2024). The program, called Kidney for Life (KFL), was created to improve the level of HLA compatibility and the long-term allograft longevity. Under the KFL initiative, NKR has already facilitated over 120 low alloimmune risk transplants with a 98% transplant rate within 12 months. Steering generally compatible but sub-optimal donor-recipient dyads towards uncoupling and entry into kidney paired exchanges represents a reasonable and efficacious approach to both maximize transplantation of difficult-to-match sensitized patients and optimizing graft longevity for patients with compatible living donor candidates.

### 6.5 Promising but not yet ready for primetime: minor histocompatibility antigen matching

Genome-wide studies have demonstrated promise in identifying specific mHAs of concern (such as LIMS1) and in producing clinically significant mHA scores (such as the allogenomics mismatch score). Combined with the reality that the plurality of graft failures appears to be associated with non-HLA genetic risks, incorporation of mHAs in donor-recipient matching and post-transplant care personalization as outlined above will likely represent a significant opportunity to improve long-term transplant outcomes. However, mHA-incorporating matching and management is not currently ready to enter the clinic, due to several technological and scientific limitations.

Further genome-wide studies will be required to establish comprehensive, accurately predictive genome-wide polygenic risk scores with high validity and clinical interpretability. Current scores are limited by either being developed on relatively small sample sizes or having strong associations with graft failure risk but unclear individual predictive value. Further retrospective and prospective studies will be required to further develop and refine these scores. The growing iGeneTrain database likely represents the best opportunity to conduct the future studies needed to generate highly prognostic mHA risk scores. Logically, a mHA score would eventually follow eplet and/or PIRCHE-II based molecular matching in some of the above applications, albeit with several additional limitations. First, currently whole-genome and whole-exome sequencing technologies remain relatively expensive, although their cost continues to decline, and they are rapidly entering the clinic for the diagnosis of suspected rare single-gene variants (Schwarze et al., 2018). It is possible that a targeted panel of selected mHAs with well-established clinical significance could be cost-effectively incorporated, even if genome-wide panels remain prohibitive. Either way, firmly establishing the clinical implications of specific mHAs and moving from genome-wide associations to clear, clinically interpretable scores will be an essential next step before prospective use of mHA-based molecular matching can be implemented.

## 7 Conclusion

Although the era of antigen-based HLA matching continues, rapidly improving sequencing technologies have created the opportunity to move beyond the imprecise serological reduction of HLA typing and base matching on the true immune-molecular reality of host-graft interaction, as well as allowing the incorporation of potentially numerous non-classical and non-HLA minor histocompatibility antigens. Although strides will need to be made to provide consistency in molecular matching, the technology has proven itself valuable in understanding disparate long-term outcomes in transplant recipients. Decoupling living donor pairs followed by entry into a paired kidney exchange presents a significant opportunity to leverage molecular matching to both optimize immunologic risk (and therefore long-term outcomes) for kidney recipients while simultaneously improving the odds of transplant for very highly sensitized patients. And for recipients of both living and deceased donor organs, molecular matching may allow for immunosuppression minimization for incidentally or intentionally optimally matched donor-recipient pairs. These approaches are ready to enter the clinic in the form of clinical trials—followed eventually by the incorporation of molecular matching in deceased donor allocation, the integration of minor histocompatibility antigens in matching, and beyond.
